# A Qualitative Study on Continuing Medical Education Programs for Practicing Ophthalmologists in Iran: Changing Previous Norms

**DOI:** 10.30476/JAMP.2021.89881.1389

**Published:** 2022-01

**Authors:** SEYED ALIAKBAR FAGHIHI, SOLEIMAN AHMADY, MASOMEH KALANTARION, AMIN HABIBI, SEPEHR FEIZI

**Affiliations:** 1 Clinical Educational Research Center, Department of Medical Education, Shiraz University of Medical Sciences, Shiraz, Iran; 2 Department of Medical Education, Virtual School of Medical Education and Management, Shahid Beheshti University of Medical Sciences, Tehran, Iran; 3 Ophthalmic Research Center, Research Institute for Ophthalmology and Vision Science, Shahid Beheshti University of Medical Sciences, Tehran, Iran

**Keywords:** Ophthalmologist, Continuing medical education, Qualitative research

## Abstract

**Introduction:**

The Continuing Medical Education (CME) programs in the medical community aim to improve the knowledge of practitioners and its effect on changing their performance. Previous studies showed that CME causes minimum changes in physicians’ behaviors, so it is important to pay attention to the views of this group. In this regard, this qualitative study aimed to explain the Iranian Ophthalmologists’ perceptions and experiences concerning the CME Programs in Iran.

**Methods:**

In this qualitative study, 18 participants, including 10 subspecialists and 8 general ophthalmologists, were recruited to participate in in-depth interviews concerning their experiences with CME. The required data were collected from April 2018 to Feb 2019. Each interview was conducted in medical universities; eye research centers; and the ophthalmology departments, offices, and operating rooms of public and private hospitals. The current study was performed using a content analysis based on the Granheim and Lundman’s methods. The codes, sub- categories, categories, and themes were then explored through an inductive process in which the researchers moved from specific to general.

**Result:**

The data obtained from interviews, and filed notes were analyzed and then classified into the following four themes: "growth and development of the ophthalmology's CME program over time", "challenges of the ophthalmology's CME program", "reasons for the participation of the ophthalmologist in the CME program", and "strategies for improvement".

**Conclusion:**

Based on the qualitative study’s results, in spite of growth and development of the design and implementation of the Ophthalmologic CME programs, we are still facing multiple challenges. Enhancing the interactivity between the providers and participants can also improve the ophthalmology CME programs.

## Introduction

Human resources are known as the most important source of health system, and performance of health care systems highly depends on the knowledge, skills, and motivation of those staff who are responsible for providing care services. Accordingly, training is a key investment tool for the obsolescence of old skills as well as the emergence of new technologies in the provision of care ( 1 , 2 ).

Nowadays, lifelong learning has become an integral part of human life. In this regard, the United Nations Educational, Scientific and Cultural Organization (UNESCO) has emphasized it at the beginning of the 21^st^ century ( 3 , 4 ). Continuous learning, along with updating information and skills on health issues that also deal with community health status, is of especial important ( 5 , 6 ).

Owners of medical sciences, and especially physicians, are among the people mostly suffering from the loss of information in the scientific field for themselves and the people in the community who benefit from their care services ( 5 ).

CME refers to a specific form of the continuing education (CE) that helps those in the medical field who maintain, develop, or increase their competence and relationships in order to provide better services for patients ( 5 - 7 ). The first CME programs worldwide and in Iran were reported in 1935 and 1991 ( 8 , 9 ), respectively.

For several years, the traditional form of CME has been holding conferences, symposiums, or workshops. However, there are many other activities that can be added to this form such as chart audit and feedback, and simulated experiences ( 7 ). However, the CME programs must be designed in a way to meet the needs of the learners ( 10 - 12 ). According to articles published in other fields of medical science, the CME programs do not actually meet the needs of their learners ( 13 - 15 ). In fact, qualitative research is known as a valuable approach used to describe life experiences, which can help to better understand human experiences. In the study, qualitative data consists of the participants' perceptions and opinions ( 16 ). 

The aim of the CME programs in the medical community is to improve the knowledge of practitioners and cause a change in their performance ( 17 ). Previous studies ( 5 , 18 - 19 ) showed that the CME provided minimum changes in physicians’ behaviors, so it is important to pay attention to the viewpoints of this group.

Up to now, no study has been conducted on continuing ophthalmology education in Iran, and most of the studies performed on general practitioners are quantitative. On the other hand, most experts believe that performing qualitative research to understand the real needs of teaching and learning, the subjects’ experiences, and mental phenomena is considered as a unique solution ( 20 ). Therefore, continuing ophthalmology education was selected to be investigated in the current study. This qualitative study aimed to explain the Iranian Ophthalmologists’ perceptions and experiences regarding the implementation of the CME Programs in Iran.

## Methods

This study was designed and implemented using a qualitative content analysis method ( 21 ).

### Participants

In this qualitative study, a total of 18 ophthalmologists, including 10 professors of ophthalmology and 8 ophthalmologists with a mean age of 45± 9 years old and a mean duration of 14±9 years in ophthalmic practice were enrolled and then interviewed. The participants were selected using purposeful sampling. The inclusion criteria including the participants in this study were continuous presence in programs, a background in policymaking and program implementation with the maximum variation in terms of job title (Professors of ophthalmology, Assistant professors of ophthalmology, Associate professors of ophthalmology, and Nonacademic ophthalmologists), and educational degree (Fellowship or General Ophthalmologist). Those cases who were unwilling to participate in the interviews were excluded from the present study. 

### Ethical consideration

The present study was approved by the Ethics Committee of Ophthalmic Research Centers, Shahid Beheshti University of Medical Sciences with the approval number of IR.SBMU.ORC.REC.1396.21. Firstly, the objectives of the study were explained to the participants prior to the data collection, and the interviews were recorded with their consent. Informed written consent was taken from all the included subjects. All the participants had the right to continue or withdraw from the study. Of note, the studied cases’ information was kept confidential. 

### Data collection

The required data were collected directly from the experiences of the study participants by holding semi-structured and in-depth individual interviews. Interviewing is a qualitative research technique that involves intensive individual conversations with a small number of respondents, with the purpose of exploring their perspectives on a particular idea, program, or situation ( 22 ). Data collection was conducted from April 2018 to Feb 2019. A member of the research team (MK) who had the experience of conducting qualitative research, was interviewed. She had participated in some ophthalmology continuing education programs and had interactions with participants, lecturers, and program providers to better understand the phenomenon of the continuing ophthalmology education. Purposive sampling was continued until data saturation, meaning that no additional information was obtained for the concept. Subsequently, categories and themes were repeated. The length of each interview varied from 40 to 60 minutes depending on the interviewee's circumstances and their agreement. We have planned to perform data collection from each participant by a single interview visit, but in some cases, there was a need to arrange additional interviews. It is noteworthy that three participants were interviewed twice. Overall, a total of 21 interviews were conducted. The interview guide was a short list consisting of some general questions such as "Please explain your experience in continuing ophthalmology education programs?" or "Describe the last time you participated in a continuing education program?". Following the interviews, follow-up questions, including "Please give an example in this case?" and "Explain more", were asked based on the participants' statements and the provided information in order to clarify the concept under the study. The following questions were based on the participants’ answers. The interviews were listened to by MK until the researcher understood their content and main ideas, and the ambiguities were clarified immediately after holding the interview through referral to the interviewee. Data were collected in those environments where this phenomenon occurred. Thus, each interview was conducted in medical universities (Shahid Beheshti University of Medical Sciences, Tehran University of Medical Sciences, Iran University of Medical Sciences, Baqiyatallah University of Medical Sciences); eye research centers; and the ophthalmology departments, offices, and operating rooms of public and private hospitals.

### Data analysis

The content analysis method suggested by Graneheim and Lundma (2004) ( 21 ) was used at this stage. After each interview, its record was immediately transcribed by MK. Thereafter, the whole text of each interview was considered as the unit of analysis. Afterward, each unit was abstracted and named with a code by two researchers (MK, AF). To find similarities and differences, MK and AF read the preliminary codes several times and then compared them. In case of any disagreement, a third researcher (SA) was involved. The sub-categories were categorized based on the similarities. Finally, the categories were developed and integrated to the themes. Of note, we used no software for analyzing the data.

### Trustworthiness

To ensure the trustworthiness of the obtained data, Credibility, Confirmability, Dependability, and Transferability criteria were used as the criteria of scientific accuracy in the qualitative research, which were firstly presented by Guba and Lincoln ( 23 ).

For credibility, the researcher spent a long time immersing in the data and transcripts, and enough time was allocated for collecting and then analyzing the information. For credibility, the researcher spent a long time on the data using note taking during the semi-structured interviews. The Dependability of the data was evaluated by the observers’ review (peer-check) and external review (member check). The initial findings of the current study were presented to some participants in the form of the codes and categories, and their comments were received (member checking) as well. Some parts of each interview were analyzed by the colleagues who were not present in the study (peer-checking), and thereafter, the findings were confirmed based on their analyses; also, the findings were repeatedly evaluated by the supervisors (expert checking). Additionally, in a meeting with the ophthalmology experts, the findings of this study were presented and then approved.

Moreover, as to the Dependability of the findings, they were presented and approved in a meeting with the ophthalmology experts and quality experts as a specialized panel.

Other evidence obtained from other studies, as well as opinions and ideas of the researchers and documentation of the study's findings helped to prove the confirmability of the data. Finally, by providing a comprehensive description of the concepts, contributors, content, data collection and analyses, methods used, and the study limitations, the transferability of the data was confirmed, so that other researchers can follow the research process employed by the researchers of this study.

## Results

The results of this study are based on the experiences and perceptions of the participants of the four main themes, including "growth and development of the ophthalmology's CME program over time", "challenges of the ophthalmology's CME program", "reasons for the participation of the ophthalmologist in the CME program", and "strategies for improvement,” which are described below, respectively ( Table 1).

**Table 1:** Themes, categories and sub-categories extracted from interviews with ophthalmologist

NO.	Categories	Sub-Categories	Code
1	Growth and development	Gradual maturity of ophthalmology's CME program over time	Planning for job promotion
			Improving the quantity and quality of programs
			Usefulness of programs in clinical work
			Facilitate access to ophthalmologists to participate in the programs
		Empowering structure	Facilities and equipment
			Human resources
2	Challenges	Dominance of income generation in designing and implementing CME programs	The preferences of ophthalmologists
			The effective role of financial sponsors in determining the content of programs
		Non-comprehensive education	Unilateralism
			One-dimensional evaluation
			Teaching old topics
		Inadequate cooperation for design and implementation of programs	Trans-part inconsistency
			Inadequate cooperation for implementation of programs
		The paternalist view	The paternalist view in design
			The paternalist view in implementation
3	Reasons for participation	Institutionalization	Acceptance of the CME programs
			Variety in teaching methods
			Acquiring scientific authority
		Motivational factors	Internal factors
			External factors
4	Strategies to improve	Content correction	Balancing surgical and internal issues
			Selecting up-to-date and efficient topics
			Performing needs assessments
			Avoiding duplicate titles
			Improving scoring methods
		Implementation Improving	Improving teaching methods
			Choose the suitable place
		Improve program evaluation	Internal and external evaluation
			Variety in the methods of evaluation
			Promotion of evaluation culture

*1. Growth and Development of the ophthalmology CME program over time:* Based on the participants' experiences, the concepts of growth and development of the ophthalmology CME program over time refer to the two basic components. Accordingly, the first one is related to the gradual maturity of the ophthalmology CME program over time, and the second one is related to the empowering structure. 

Based on the participant's experiences, the gradual maturity of the CME program includes the following factors: planning for job promotion, improvement of both quantity and quality of the programs, usefulness of the programs in the clinical work, and the facilitation of having access to the ophthalmologists to participate in these programs.

In this regard, one of the included participants stated that:

* "Currently, our scientific capabilities and facilities are such that we can hold a regional congress or even an international one. The content of the programs is also excellent and up-to-date. Speakers mostly try to find and present the latest and most important papers compared to the past."* (P1) 

Empowerment in terms of the facilities, equipment, and human resources was another factor found to affect both the growth and development of the CME program, so we created the category of empowering structure for designing as well as implementing the ophthalmology training programs. An ophthalmologist described the empowerment of human resources as follows: *"...Another point that is very important and effective in conferences is the existence of stable executive staff, which has made them more coordinated and empowered."* (P18)

Another ophthalmologist expressed her opinion on being empowered in terms of the facilities and equipment as below: 

*"...The form of presentation has become more modern, and smarter tools are used more nowadays; also, live surgery is another thing practicing now that has not been done before..."* (P6).

*2. Challenges of the ophthalmology' CME program:* According to the perceptions of the included participants, the CME programs also have some challenges; therefore, paying enough attention to them and then eliminating them will help making these programs better. This theme can be described by four related categories, including "dominance of the income generation in designing and implementing the CME programs," "non-comprehensive education," "inadequate cooperation for the design and implementation of these programs", and "the paternalist view."

Moreover, "the preferences of the ophthalmologists" and "the effective role of the financial sponsors in determining the content of programs" were among the sub-categories clarifying the role of the income generation dominance in both designing and implementing the CME programs. 

Earning more money was introduced as one of the ophthalmologists' preferences for participating in the CME programs. 

In this regard, a participant stated:

*"…Most of the ophthalmologist likes to participate in the surgical ophthalmology seminar in comparison with dry eyes seminar because of the labor market. So, the content and topic of the programs are more inclined toward the surgical ophthalmology due to their interest…."* (P16).

Non-comprehensive education was also identified as one of the challenges of implementing the CME program. Accordingly, the ophthalmologists defined this category using the concepts of "unilateralism," "one-dimensional evaluation", and "teaching old topics."

For example, one participant explained the concept of unilateralism as follows:

*"…The problem is that the content of programs is mainly based on the perspective of subspecialists. The content should be based on the perspective of general ophthalmologists as well because they account for the majority of the ophthalmologists' population. Perhaps, a simple matter is obvious for the specialty, but that matter may not be obvious to general ophthalmologists…."* (P14).

"Inadequate cooperation for the design and implementation of the programs" was another stated challenge in the Ophthalmology CME program as it was emphasized by one of the participants as follows: 

*"For example, a speaker has 60-100 slides to present in 10 minutes. He cannot present all of them, so he has to skip some of the slides; on the other hand, the judges warn him, and this makes us not understand that presentation, and generally, it consequently causes chaos."* (P3)

Ophthalmologists also believed that the dominance of the expert ophthalmologists in designing most of the programs against the needs of the participants and the consistency amongst the guides, speakers, and chairmen of the congressional panels demonstrate the dominance of the paternalist views. In this regard, one of the included ophthalmologists said, *"…The expert's opinion is always dominant; for example, the titles of seminars and congresses are always selected by experts and not based on the needs of all participants…."* (P15).

*3. Reasons for the participation of the ophthalmologist in the CME program:* The way of the ophthalmologists' participation in the CME programs involves a range, and some factors such as learning, improving the performance, or just earning the related points could affect it. This theme is described by the relevant concepts of the institutionalization and motivational factors. With regard to the concept of the institutionalization, one of the participants said:

*"…Today, in our country, participation in educational programs is institutionalized. This is a very good event, so that every doctor knows that in order to renew his office license, he/she needs to participate in these kinds of programs and earn the points…"* (P12).

Motivational factors (including the internal and external ones) are known as another factor affecting the ophthalmologists' participation in the CME programs. Many ophthalmologists stated the reasons for their participation in the CME programs related to the internal factors, such as personal motivation and their scientific needs. Moreover, scoring points to extend the office licenses, creating a competitive atmosphere for earning the points, running the programs in the form of group discussions, updating the topics, and helping the generation of more revenue were some of the factors stated by the participants in the current study as the external factors affecting the participation in the CME programs. 

*4. Strategies for Improvement:* Most of the enrolled ophthalmologists suggested some ways to increase the effectiveness of the CME programs for the learners, which leads to their greater participation in these programs. In this study, this theme was described by the following categories: content correction, improvement of the implementation of the CME programs, and improvement of the program’s evaluation.

Of note, the strategies for correcting the content of the ophthalmology CME program were among the solutions mentioned by the participants in this study. The ophthalmologists believed that the CME programs could be upgraded by making a balance between the surgical and internal issues, selecting some up-to-date and efficient topics, performing the needs assessments, avoiding from the presentation of the duplicate titles, and improving the scoring methods.

An ophthalmologist stated:

*"…I think there is a problem in our health policy system and believe that internal medicine should be considered along with surgical procedures because, for example, if you treat a patient with the dry eyes and cataracts by the phacoemulsification surgery, then you will face lots of problems later, so these issues are interconnected, and a balance must be made between them…."* (P4).

Improving the implementation of the CME programs was another solution mentioned by many participants. Correspondingly, one participant stated:

*"…Recently, we have had several programs regarding virtual training, which is very good because it leads to a constant presence of the audience and has no spatial or temporal limitations…."* (P2).

The need to improve the program evaluation emphasize by most of the participants in the study. Providing feedback on the evaluation results to the ophthalmologists leads to the promotion of a culture of participation. As an ophthalmologist put it: *"…Evaluation is very important. We can recognize a good speaker or organizer through proper evaluation. Unfortunately, the results of the program evaluation forms don't report anywhere. Therefore, our motivation to participate in program evaluation has decreased ..."* (P11).

## Discussion

The present study aimed to explain the understanding of Iranian Ophthalmology Association on how to provide the CME plans. In this study, the structure of the included participants' experiences of participating in the CME plans was classified into the following four major themes: "growth and development of the ophthalmology's CME program over time", "challenges of the ophthalmology's CME program", "reasons for the participation of the ophthalmologist in the CME program", and "strategies for improvement". 

The concepts of growth and development of the ophthalmology CME program over time pointed out the two basic components of gradual maturity of the ophthalmology CME program over time as well as empowering structure. According to the results of the study, the Scientific Association of Ophthalmology and Scientific Secretaries, by considering the academic approach, introduced a professional approach for the CME programs for job promotion of ophthalmologists. Pendleton also mentioned academic and professional approaches to CME as different from each other. The physicians practicing medicine are more likely to benefit from education compared to when they are in a CME program. However, many CME approaches are not designed based on the academic model and implemented due to physicians' clinical problems or are very little related to the physicians' clinical problems ( 24 ).

In terms of facilities, equipment, and human resources in the present time compared to the past time, empowerment was found as one of the concepts that clarified the nature of the empowered structure for designing and implementing the ophthalmology education plans.

Ophthalmologists' ability to use equipment has increased, and making foreign companies allocate facilities to plans has been observed as an excellent opportunity. In a study conducted at Tehran University of Medical Sciences, the general practitioners and specialists considered the presentation of new scientific achievements, professors’ capabilities, new educational methods, and interactive education sessions as the prominent points of the CME plans ( 25 ).

Resolving the challenges of the ophthalmology CME programs can help improve these plans. The challenges of the plan are described by four related concepts as follows: the dominance of income generation in both designing and implementing the CME programs, non-comprehensive education, inadequate cooperation for the design and implementation of programs, and the paternalist view. 

Most of the participants in this study believe that besides the risk of surgery and its side effects, earning more money from these surgeries has also led to a greater preference for physicians to participate in continuing surgical education plans. Other studies also reveal that physicians avoid any program that spends more work time or the one that does not increase their income ( 26 ). 

The ophthalmologists believed that CME plans were not comprehensive in terms of the content and implementation. They defined non-comprehensive education with the concepts of unilateralism, one-dimensional evaluation, and teaching old topics.

According to the results of this study, policy makers should take a more serious look at evaluation and perform it in different ways, which are not currently the case.

In this study, it was found that the repetition of routine issues in plans as well as the lack of proposing up-to-date plan topics in terms of content along with the up-to-date needs assessment led to the teaching of old topics in some of the CME plans. A study by Navabi et al. showed the repetition of plan topics ( 27 ). Moreover, in a study by Bauer et al., "subject of the plan" gained the highest importance (81%) from the perspective of the participating dentists ( 28 ).

The lack of proportionality between the number of participants and the announced capacity has led to the neglected educational purposes. The participants also believed that non-readiness of some speakers to present, failure to discuss and answer questions, and lack of time had led to chaos in this regard. A study by Navabi et al. also indicated that speakers should spend more time on designing their lectures, so that the lecture contents are different from what are taught to Ph.D. students ( 27 ).

Other results of this study include the paternalist view in the design and implementation stages. For explaining this issue, we can say that during the processes of designing and implementing plans, we are usually facing the phenomenon of patriarchy. Experts are the ones who determine what should be thought to the general practitioners. In this regard, Stewart and Khadra in their study have acknowledged the existence of the paternalist spirit in the design and presentation of content to physicians in the CME plans ( 29 ).

The concept of reasons for the participation of the ophthalmologist in the CME program includes both conditions and situations that are effective in directing the behavior and decision of an ophthalmologist to participate in the plan. Correspondingly, this theme is described by the two relevant concepts of institutionalization and motivational factors.

The acceptance of the program, variety of educational methods, and acquisition of scientific authority have led to the institutionalization of the CME programs among the ophthalmologists. According to most of the enrolled participants, the earlier start of the CME plans compared to neighboring countries and having the most significant number of participants in these programs in the region have made the ophthalmology department a recognized scientific authority in this region. Meanwhile, in Pakistan, CME is still a relatively new concept, and very few physicians regularly attend its sessions ( 30 ).

Ophthalmologists' presence and participation in the CME plans were indicated to be affected by various factors, including internal and external motivational factors. In this regard, the internal motivational factors include the personal motivation and scientific needs of ophthalmologists. In a small number of previously performed studies, the most important motivation to participate in the plan was for gaining the points. In most of these studies ( 31 , 32 ), the most important motivation for participating in the program was reported to be the professional need of the participants ( 33 - 37 ).

On the other hand, the results of this study show that ophthalmologists participate in plans with surgical subjects because of the prevalence and importance of this issue; further income generation; and more practical, urgent, and stressful surgical cases. The general practitioners and specialists in Yazd Province have introduced the teaching of new practical skills as their major motivation to participate in the plans ( 35 ).

Despite the growing trend of ophthalmology CME plans, there are still some challenges in these plans, some of which were mentioned earlier. Of note, most of the included participants suggested some ways to increase the effectiveness of CME.

The results of the study indicate that a successful CME needs assessment should be performed appropriately; also, seminars should be held by a trustee that leads to the prevention of repeated subjects and speakers and parallel works. Studies conducted on the factors related to needs assessment showed that most general practitioners and specialists called for seasonal and local discussions select subjects based on surveys ( 38 - 40 ). 

Correcting the scoring method in such a way that ophthalmologists' performance in the CME plans can also be scored, and allocating resources in a balanced way are the other results of this study based on the participants' experiences. Florence et al. in their study showed that retraining scores should not only be considered for participating in plans, but also for using some criteria to evaluate the effectiveness of the plan such as testing at the end of retraining or after it for examining the physicians' performance in post-retraining ( 41 ).

Additionally, improvement of the implementation of CME programs is an issue that affects all other factors related to education plans at all stages of a CME plan from the start to the end. 

Ophthalmologists believe that the form of presentation of some professors should be corrected. Furthermore, the familiarity of ophthalmologists with medical education can consequently improve the presentation of lectures. Kousha et al. also suggested that professors teaching CME plans should be trained in the field of the teaching process, new teaching stages and patterns, characteristics of adult learners, and learning theories in a workshop. One of the reasons for professors' use of lecturing methods is their lack of familiarity with the use of new teaching methods and patterns, especially participatory methods ( 41 ).

One of the results emphasized by the participants was the need to improve the program’s evaluation. It can be done through internal and external evaluations, variety in the evaluation methods, and promotion of evaluation culture. Changes in the approach of evaluation and the use of combined methods lead to more participation of individuals in the evaluation ( 42 ). A group of experts believed that some variables such as education methods, learning methods, and environment were effective on learning, so it is not possible to examine all aspects of the processor’s competence by performing only one type of evaluation ( 43 ).

## Limitations and weaknesses

One of the limitations of the present research was that some participants did not allow us to record their conversations; therefore, the researcher had to write down everything she heard. Another restriction was that the researcher made several appointments to interview the participants, but the interviews were delayed due to the ophthalmologists' busy schedule.

## Conclusion

Based on the results of the current qualitative study, influential parameters affect the effectiveness of the Ophthalmologic CME programs in Iran. Despite the growth and development of both the Ophthalmologic CME programs' design and implementation, we still face multiple challenges and weaknesses, including lack of coordination between CME programs and the field of activity. Besides the insufficient attention paid to professional demands and educational content, paternalistic view in design and implementation should also be considered. Enhancing the interactivity among providers, trainees, and trainers can consequently improve CME programs, thereby enhancing the performance of ophthalmologists during the time. A grounded theory approach is suggested for comprehensive exploring the ophthalmology CME process in Iran.
